# A novel exopolysaccharide-producing and long-chain n-alkane degrading bacterium *Bacillus licheniformis strain* DM-1 with potential application for *in-situ* enhanced oil recovery

**DOI:** 10.1038/s41598-020-65432-z

**Published:** 2020-05-22

**Authors:** Yanhui Fan, Jun Wang, Chunming Gao, Yumiao Zhang, Wen Du

**Affiliations:** 1 College of Biological and Environmental Engineering, Binzhou University, Binzhou, 256600 P.R. China; 2 Shandong Engineering and Technology Research Center for Wild Plant Resources Development and Application of Yellow River Delta, Binzhou, 256600 P.R. China; 3 Shandong Provincial Key Laboratory of Eco-environmental Science for Yellow River Delta, Binzhou, 256600 P.R. China

**Keywords:** Environmental biotechnology, Industrial microbiology, Microbiology, Applied microbiology, Environmental microbiology

## Abstract

A novel *Bacillus licheniformis* strain (DM-1) was isolated from a mature reservoir in Dagang oilfield of China. DM-1 showed unique properties to utilize petroleum hydrocarbons and agroindustrial by-product (molasses) for exopolysaccharide (EPS) production under oil recovery conditions. The DM-1 EPS was proven to be a proteoglycan with a molecular weight of 568 kDa. The EPS showed shear thinning properties and had high viscosities at dilute concentrations (<1%, w/v), high salinities, and elevated temperatures. Strain DM-1 could degrade long-chain n-alkanes up to C36. Viscosity reduction test have shown that the viscosity of the crude oil was reduced by 40% compared with that before DM-1 treatment. Sand pack flooding test results under simulated reservoir conditions have shown that the enhanced oil recovery efficiency was 19.2% after 7 days of *in-situ* bioaugmentation with *B. licheniformis* DM-1. The obtained results indicate that strain DM-1 is a promising candidate for *in situ* microbial enhanced oil recovery (MEOR).

## Introduction

Two-thirds of crude oil remains entrapped in oil reservoirs even when it is subjected to routine water and gas flooding^1,2^. Effective exploitation of these untouched crude oil is critical to meeting the world’s growing energy demands, and attention has been focused on tertiary oil recovery technology to enhance oil recovery (EOR) to produce more crude oil in the past decades^3,4^. Polymer flooding is one of the most widely used techniques in tertiary oil recovery. The mechanism of polymer flooding is to add a high-viscosity water-soluble polymer into the injected water to expand the swept volume and improve mobility ratio, thereby enhancing the oil recovery^5,6^. Sheng *et al*.^7^ collected and surveyed 733 polymer-flooding projects in 24 countries worldwide and concluded that the median incremental oil recovery is approximately 6.7%.

Nowadays, partially hydrolyzed polyacrylamide and its derivatives are the most widely used substances in polymer flooding and have been used in a lot of oil fields for large-scale oil production^8,9^. However, these chemical synthetic polymers that are toxic to the environment and difficult to decompose^6^. Therefore, biologically produced polymers (biopolymers) have attracted increasing attention because they are eco-friendly and have superior tolerance to salts and temperature^10^.

More than 10 biopolymers, including schizophyllan, xanthan, guar gum, hydroxyethylcellulose, scleroglucan, carboxymethycellulose, and lignin, have been reported for EOR field studies. The field application of schizophyllan flooding in Bockstedt oil field (Germany) has shown that the oil production rate increases by more than 20% compared with that of waterflooding^11^.

Two main strategies are available for the use of biopolymer in MEOR: 1) *ex-situ* where biopolymer is directly injected into the oil reservoir with the injected water and 2) *in-situ* where suitable microbes are injected to produce the desired biopolymer in the oil reservoir. In most cases, biopolymer flooding is carried out via the first strategy. For example, xanthan gum (obtained from *Xanthomonas campestris* pv. campestris), the most widely known and used biopolymer, is normally produced *ex-situ* and injected into oil reservoirs with the injected water to EOR. However, the *ex-situ* process, for example, determines optimal fermentation conditions, purifies the EPS, transports and injects it into the well, and leads to high production costs, which limits its use in the oil field^12^. In contrast, the *in-situ* process that injects suitable microorganisms into the oil reservoir to produce the desired polymer seems to be cost-effective. For example, BNP29, a strain of *B. licheniformis* isolated from a German oil reservoir, can improve oil recovery efficiency by up to 22.1% after *in-situ* microbial treatment^13^, and ZR3, an engineered strain constructed from a EPS-producing strain and a thermophilic strain, can improve oil recovery by up to 11.3% of the OOIP over water flooding after 7 days *in-situ* bioaugmentation^14^.

Many depleted and mature reservoirs are available in China that need to be rejuvenated. However, few researches have investigated indigenous microorganisms in local oil fields for MEOR. In this study, an EPS-producing strain (DM-1) was isolated from oil field-produced water, and its EPS production capacity under extreme environmental conditions was studied. Sand pack flooding tests were performed to determine the effects of *in-situ* bioaugmentation with *B. licheniformis* DM-1.

## Materials and Methods

### Crude oil and reagents

Crude oil with a viscosity (50 °C) of 110 mPa·s and a density of 0.87 g/cm^3^ was obtained from Dagang Oilfield. Analytical-grade chemical reagents were used in the experiments.

### Microorganism

**Figure 1:**
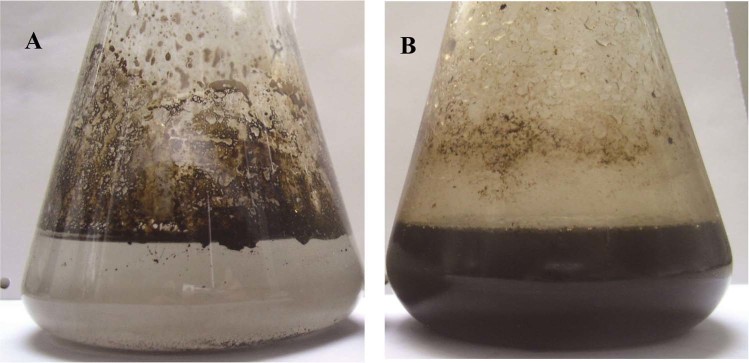
Ability of *B. licheniformis* DM-1 to degrade crude oil. (**A**) Crude oil in BH medium without inoculum of DM-1. (**B**) Crude oil in BH medium inoculated with DM-1. Results were obtained at 55 °C for 7 days.

Strain DM-1, which can degrade and emulsify crude oil (Fig. 1), was isolated from a mature reservoir in Dagang oilfield of China and deposited in China General Microbiological Culture Collection Center with the deposit number of CGMCC 1.6128. Pure culture of DM-1 was preserved in sterile glycerol suspensions (20%, v/v) at −80 °C; for routine experiments, DM-1 was maintained on LB agar slant at 4 °C and subcultured at an interval of 30 days.

### Identification of strain DM-1

DM-1 was identified based on physiological and morphological characteristics and 16 S rRNA sequence analysis. Chromosomal DNA was extracted from *B. licheniformis* DM-1 by using a Bacteria DNA Kit (Takara Bio Inc.) according to the manufacturer’s instructions. The 16S rRNA gene sequence was amplified by using the universal primers 27 F (5'-GAGAGTTTGATCCTGGCTCAG-3') and 1541 R (5'-AAGGAGGTGATCCAGCCCGCA-3'). The purified amplified product was sent to Takara Bio Inc. (Dalian) for sequencing. The 16S rRNA gene sequence was used to search the GenBank database (https://blast.ncbi.nlm.nih.gov/Blast.cgi) with the BlastN algorithm and determine the relative phylogenetic position. Phylogenetic analysis based on neighbor-joining method^15^ was performed by using MEGA 6.0^16^. The stability of relationships was assessed by performing bootstrap analyses of the neighbor-joining data based on 1,000 resamplings.

### Exopolysaccharide production

Active bacterial culture was prepared by transferring one loop of strain DM-1 from agar slant into 10 ml of LB medium and incubated at 45 °C at 180 rpm for 12 h. The bacterial cells in the culture broth were collected via centrifugation at 6,000 rpm for 10 min. Thereafter, bacterial cells were washed thrice with sterile water and were used to inoculate 100 ml sterilized Bushnell–Haas (BH) medium (KH_2_PO_4_ 0.1%, CaCl_2_ 0.002%, NH_4_NO_3_ 0.1%, MgSO_4_·7H_2_O 0.02%, K_2_HPO_4_ 0.1%, FeCl_3_ 0.005%, pH 7.0) supplemented with petroleum or nonpetroleum carbon sources, such as crude oil, liquid paraffin, starch, glucose, sucrose, and molasses (2%, v/v) or (2%, w/v). Nitrogen was injected to replace the air in the flasks^14^, and the flasks were sealed with non-vented rubber stoppers after inoculation of strain DM-1 into the Erlenmeyer flasks to create anaerobic environmental conditions. The parameters studied for EPS production included salt concentration (0, 1, 3, 5, 7, 9, and 11%, w/v of NaCl) and varying temperatures (30 °C, 40 °C, 45 °C, 50 °C, and 55 °C). Strain DM-1 was routinely cultured in 300 ml flasks, and culture broth was taken at intervals to analyze EPS production and bacterial cell growth (OD_600_).

### Preliminary chemical characterization of the exopolysaccharide

The strain DM was cultured under optimal conditions (BH medium supplemented with 2% sucrose, 5% (w/v) NaCl, 45 °C; 60–70 h of incubation), and then EPS was purified and analyzed to determine the nature of EPS. The method of polymer purification was according to the method of Ortega–Morales *et al*.^17^ with slight modifications. In brief, bacterial cells were removed through centrifugation at 10,000 × *g* and 4 °C for 20 min and filtered through HVLP filters with a pore diameter of 0.47 µm. The supernatant was kept at 4 °C overnight, and EPS was precipitated by adding two volumes of cold absolute ethanol. The precipitate was recovered through centrifugation at 120,000 × *g* and 4 °C for 20 min, dissolved in a small volume of distilled water, subsequently dialyzed against deionized water for 48 h, reprecipitated, and dried at 40 °C. The total carbohydrate content was evaluated by phenol–sulfuric acid method described by Dubois *et al*.^18^. Protein content was determined by using the Lowry method^19^. Bovine serum albumin (BSA) was used as the calibration standard. The lipid content was determined by using gravimetric estimation^20^. The sugar components and proportions of the purified EPS were investigated by using HPLC method^21^. The functional groups in the bioemulsifier were analyzed by Fourier transform-infrared spectroscope.

### Physicochemical characterization of the DM-1 EPS

#### Rheological characterization

The rheological properties of the DM-1 EPS, at concentrations ranging from 0.25% to 0.75% (w/v), were measured at 25 °C at a shear rate of 20 s^−1^ using a digital rotational viscosimeter (BROOKFIELD, LVDV-II + Pro, USA). EPS solutions (1.0%, w/v) were incubated in a water bath at 100 °C for 10, 20, 30, 40, 50, and 60 min to investigate thermal stability, and the viscosity of the solution at the corresponding temperature was measured. Moreover, the viscosity of xanthan gum (1.0%, w/v) was tested under the same conditions. The effect of salinity on viscosity activity was determined by adjusting the concentration of added NaCl to 1%, 3%, 5%, 7%, 9%, 11%, 13%, and 15%, w/v of NaCl. The sample was allowed to equilibrate for 2 min before the viscosity was determined. Values presented are the mean of triplicate analyses.

#### Degradation of n-alkanes

The ability of DM-1 to degrade n-alkanes was tested according to a modified method described by Zhou *et al*.^22^. Briefly, inoculum (2%, v/v) of *B. licheniformis* DM-1 was added to 100 ml BH medium supplemented with 1% (w/v) individual n-alkanes (C8–C40) in 300 ml Erlenmeyer flasks. The uninoculated flasks were used as controls. The flasks were sealed with non-vented rubber stoppers and incubated for 10 d at 45 °C. Thereafter, the residual alkanes in the culture broth were extracted with 300 ml n-hexane. The alkane extracts were then analyzed via gas chromatography (GC) analysis, and their relative abundances were calculated^23^. GC analysis was performed on an Agilent 6820 machine by using an FID detector (Agilent 6820, United States) equipped with an HP-PONA column (50 m × 0.2 mm × 0.5 mm). The column temperature was kept at 120 °C for 2 min and then raised to 300 °C at a rate of 5 °C/min. Nitrogen was used as a carrier gas.

#### Reduction of oil viscosity

The oil degradation characteristics of DM-1 strain prompted us to study the potential role of DM-1 in the viscosity reduction of crude oil. The property of viscosity reduction of crude oil by *B. licheniformis* DM-1 was studied by the method of Zhou *et al*.^22^.

In brief, DM-1 strain was cultured in an LB medium and incubated at 45 °C and 180 rpm for 14 h. Then, 30 ml of the culture broth and 30 g of crude oil were added to 300 ml Erlenmeyer flasks. The flasks were sealed with rubber stoppers and incubated at 45 °C for 15 days. The sample was heated to 80 °C and then centrifuged at 12,000 × *g* for 10 min for dehydration. Oil viscosity was also measured at 50 °C by using a Brookfield viscometer (LVDV-II + Pro, USA).

#### *In-situ* microbial oil recovery

The potential application of strain DM-1 for *in-situ* MEOR was investigated by using the sand pack flooding method^23^. Sand samples (40–60 mesh), obtained from Dagang Oilfield, were washed initially with chloroform to remove crude oil and then with deionized water. They were dried at 90 °C for 24 h and filled into stainless steel columns to prepare sand pack models. The columns was 36 mm in diameter and 600 mm in length. The column permeability and porosity were 3.22 μm^2^ and 23.2%, respectively. All the sandpacks in the setups were placed horizontally. The column was saturated by injecting formation brine from the specified reservoir. The physical and chemical parameters of formation brine are listed in Table S1. The flow rate of flooding was set at 2 ml/min. Crude oil was filled in a tank and injected into the sand pack to replace the brine. The initial oil saturation was judged by measuring the volume of brine replaced by oil saturation, which is also called OOIP. After aging for 24 h at 45 °C, brine was injected (first water flooding) until no oil was monitored in the other end of the cores which means that the core reached its residual oil saturation. Thereafter, 0.35 pore volume (PV) of nutrient (BH medium supplemented with 2.0% molasses) mixed with 0.15 PV of microbes (10^9^ cells/ml) were injected into the water-flooded core, and shut-in at 45 °C for 7 days. Finally, brine was injected again until no more oil was observed in the outlet of the cores, and the amount of oil released was carefully recorded. The blank experiments were performed in the same conditions, but no bacterial cells were injected into the cores. The controls with only brine nutrients or DM-1 were performed under the same conditions to background information.

## Data analysis

All analyses in this article were carried out in triplicate, and results were statistically analyzed by using SPSS 16.0.

## Results and Discussion

In case of *in-situ* MEOR, the severe reservoir conditions (high temperature, anoxic, and hydrocarbon toxicity) affected the growth of microbes, thus influencing the efficiency of oil recovery^24^. Therefore, searching suitable microbes that can grow in oil reservoir and produce the products needed for oil recovery are necessary^25,26^. Related studies have shown that the isolation of indigenous bacteria from the target oil fields is a good method because they have adapted to the conditions of that reservoir^3^. A total of six hydrocarbon-degrading strains were isolated from a deep oil reservoir in northern China. Out of these isolates, strain DM-1 was selected for further investigation because it showed unique superiority to utilize crude oil for production of EPS under extreme environmental conditions.

### Characterization of strain DM-1

**Figure 2:**
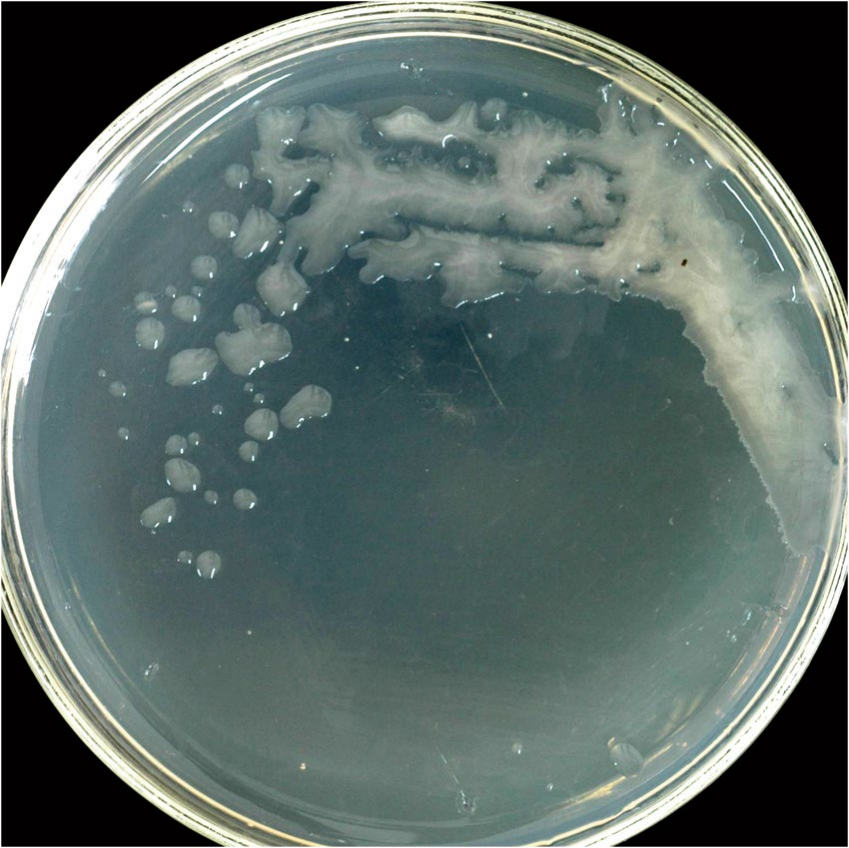
Mucoid colonies of *B. licheniformis* DM-1 on LB agar at 55 °C for 24 h.

Strain DM-1 was able to grow at 15 °C to 55 °C (optimum 45 °C), pH 6–10 (optimum 6.0–7.5), and NaCl concentration 0%–11% (w/v) (optimum 4%–5%). The ability to produce EPS was suggested by its sticky mucoid colony morphology (Fig. 2) Strain DM-1 was rod-like, facultative anaerobic, Gram-positive, and spore-forming, similar to the *Bacillus* species. The 16S rRNA gene sequence (1,541 nucleotides) was deposited in GenBank (accession number DQ539620) and compared with its database. Alignment of the 16S rRNA gene with the closest type strains showed 99.9% similarity to *B. licheniformis* strain NCTC8721 (accession number LR134165). Strain DM-1 was identified as a type of *B. licheniformis* based on physiological characteristics and 16S rRNA gene analysis (Fig. S1).

### Productivity studies

**Figure 3:**
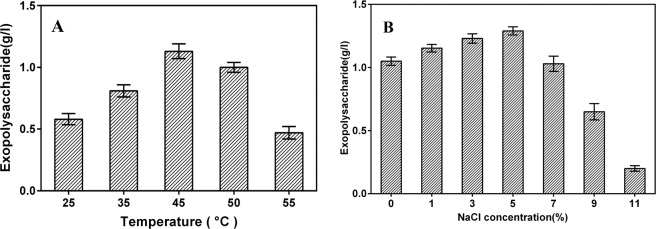
Effect of (**A**) temperature and (**B**) salinity on EPS production of *B. licheniformis* DM-1. Cultivation was carried out under optimal conditions (BH medium supplemented with 2% sucrose and 5% w/v NaCl; 45 °C) unless stated otherwise.

**Figure 4:**
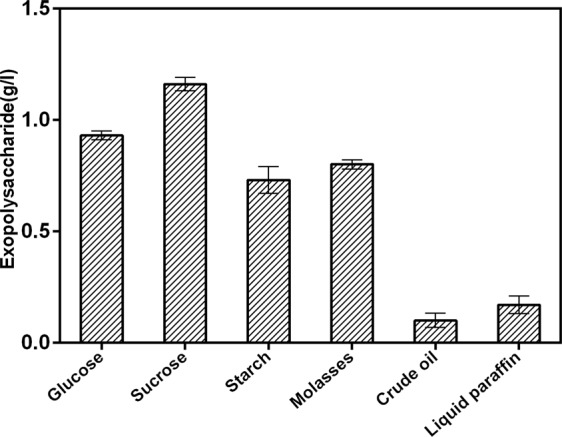
Effect of carbon sources on EPS production of *B. licheniformis* DM-1. Cultivation was carried out at 45 °C with a salinity of 5%.

The nature and quantity of EPS synthesized by a microorganism are genetically determined, but they are greatly influenced by cultivation conditions and components of medium, particularly carbon sources^27^. The effects of the cultivation conditions (temperature and salinity) on the growth of strain DM-1 and EPS yield are shown in (Fig. 3). Maximum EPS yield was achieved by incubation at 45 °C for about 60 h. The salinity in the range of 0%–7% has minimal effect on the growth of DM-1 and the yield of EPS, but the EPS yield was significantly reduced (0.2 g/l) when the NaCl concentration reached 11%. This result was consistent with several previous reports^24^. Six different carbon sources were examined for their effectiveness on EPS cproduction of strain DM-1 in BH medium with C-sources (Fig. 4). The highest DM-1 EPS production was 1.29 g/l when sucrose was used as the source of carbon. In EPS production, sugars are often the most common and effective carbon sources^28^. However, cheaper substrates (molasses, cheese whey, and glycerol byproduct) were sufficient to produce several bacterial EPSs^29–31^. The choice of cheap raw materials is an important way to reduce the production cost as they account for 50% of the final production expenses and also reduce the cost of waste disposal^28,32^. The ability of using agroindustrial by-product (molasses) to produce EPS makes DM-1 economic valuable in oil fields that require large amounts of EPS for flooding^12^.

Strain DM-1 showed unique properties to utilize hydrocarbons for the production of EPS, although the yield was not very high. This property is beneficial to the survival of DM-1 in the reservoir because crude oil is the most available and abundant carbon and energy sources in oil reservoirs^33^. The ability to utilize crude oil also makes DM-1 a potential agent for bioremediation of hydrocarbon contaminated sites, especially under *in-situ* conditions and extreme environments^34^. Accounting to relevant literature, many strains of *B. licheniformis* are able to grow and produce exopolymers, especially biosurfactants, at elevated temperature and high salt concentrations^35,36^. However, their ability to degrade long-chain alkanes and enhance oil recovery has rarely been reported^37^.

**Figure 5:**
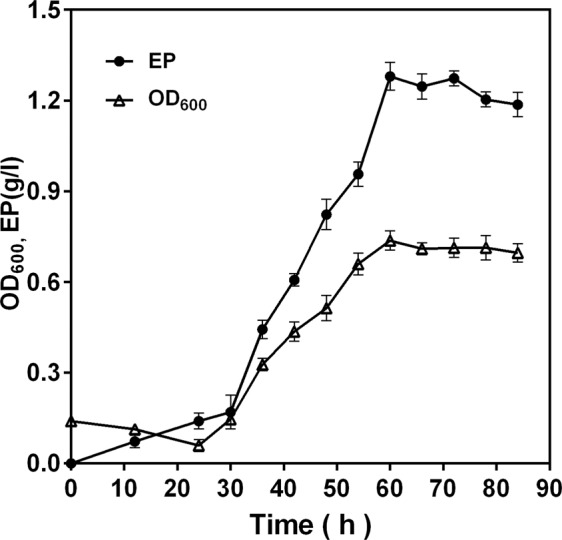
Time-course curve profile of EPS production (■) and bacterial growth (△) of *B. licheniformis* DM-1 on BH medium supplemented with 2% sucrose as carbon source at 45 °C.

The production of EPS is often associated with the growth phase of bacteria. Some EPSs are only synthesized in the late logarithmic or stationary growth phase, while others are synthesized throughout the microbial growth process^38^. Figure 5 showed the dynamic relationships between bacterial cell growth (OD_600_), incubation time, and the EPS yield under optimal conditions (BH medium supplemented with 2% sucrose, 5% w/v NaCl, 45 °C, pH 7.0). The DM-1 EPS began to produce in the early growth stage, increased with the rise of bacterial cells and reached the highest value after about 60 h of incubation at the beginning of the stationary growth stage. Afterwards, EPS yield began to decline due to enzyme activity and cell lysis^39^. The maximum polysaccharide production was 1.29 g/l after 60 h of incubation under the culture conditions described above (Fig. 5).

### Characterization of the EPS from strain DM-1

Results of FT-IR spectra analysis of the DM-1 polymer are shown in Fig. S2. The absorption peaks at 3422.41 and 1406.17 cm^−1^ were characteristic absorption peaks of O-H and C-O, which were caused by C-H and C-O stretching vibration, respectively. The peak at 2933.13 cm^−1^ was related to C-H stretching vibration. The absorption near 1600 cm^−1^ was the C-O-C stretching vibration. Furthermore, the weak peak at 780.42 cm^−1^ indicated that the sample contains β-D-type pyranose.

Compositional analyses revealed that the polysaccharide produced by DM-1 primarily consist of carbohydrate with relative percent of 67.4% sugar (w/w) and 27.6% (w/w) protein. HPLC analysis showed that the sugar fraction of the purified EPS contained an unusually large amount of mannose which accounted for approximately 91.87% of the total sugar weight (Fig. S3). Additionally, the other two sugars are glucose and galactose, which account for 7.95% and 0.18% of the total sugar weight, respectively. The protein of fraction of DM-1 EPS is mainly consist of Arg, Met, Ile, His and Lys identified by the paper chromatography method (Fig. S4).

### Rheological properties of DM-1 polymer

**Figure 6:**
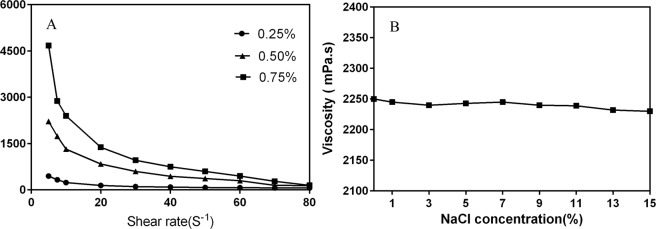
Effect of concentration, shear rate and salinity on the viscosities of DM-1 EPS.

The viscosity of EPS at concentrations of 0.25%, 0.5%, and 0.75% at different shear rates were shown in Fig. 6. The DM-1 EPS showed a comparatively high viscosity at dilute concentrations. The viscosity value of 0.75% (w/v) EPS solution was 4672 mPa·s at 25 °C at a shear rate of 7.5·s^−1^. The viscosity of the EPS solution dropped sharply when the shear rate increased from 5.0 s to 80 s^−1^, indicating that the DM-1 EPS solution belonged to non-Newtonian behavior fluid. This shear thinning behavior of the EPS solution is mainly caused by its low relaxation state and destruction of the EPS structure^40^. The effects of concentration salinity on the viscosity of DM-1 polysaccharide showed that the DM-1 EPS solutions have high viscosities at each concentration tested, and increasing salt concentration scarcely reduced the viscosities of DM-1 EPS solutions.

**Figure 7:**
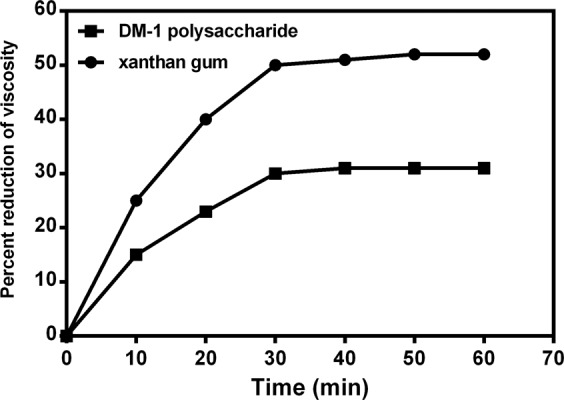
Effect of heating on the viscosities of xanthan gum and DM-1 EPS. The percent reduction in viscosity at 100 °C of a 1% (w/v) xanthan gum (●) or DM-1 EPS (■) was measured at 10-min intervals.

The rates of viscosity decrease at 100 °C of xanthan gum and DM-1 EPS dissolved in distilled water was compared. The viscosity of the xanthan gum solution decreased more rapidly than did the viscosity of the DM-1 polymer solution. After 30 min at 100 °C, about a 50% decrease in viscosity (from 11506 to 5712 mPa·s) was observed for xanthan gum compared with about a 30% decrease in viscosity (from 6862 to 4795 mPa·s) for DM-1 EPS (Fig. 7). Thermal degradation of xanthan gum at 60 °C and 75 °C in distilled water has been previously observed^35,41^.

### Utilization of n-alkanes (C12–C36) by *B. licheniformis* DM-1

**Figure 8:**
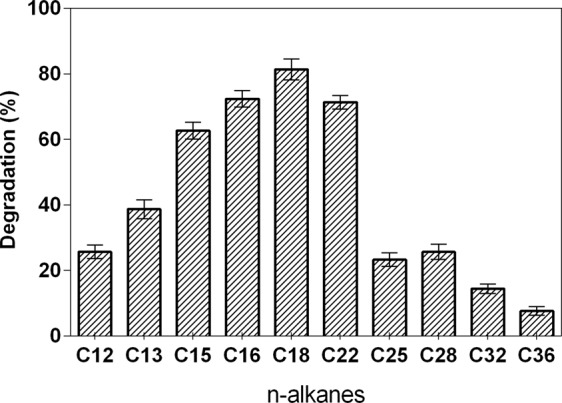
Degrading rate of the individual n-alkanes by *B. licheniformis* DM-1 over a period of 10 d (mean of three replicates; error bars represent s.d.).

Saturated alkanes (n-alkanes) are the main constituents of crude oil and are potentially the most available and abundant carbon and energy sources in oil reservoirs. Degradation of n-alkanes, especially long-chain alkanes, is of crucial importance for *in-situ* MEOR^42^. GC analysis results showed that strain DM-1 degraded a wide range of n-alkanes (C12–C36+), and preferentially degraded n-alkanes (C16–C22) with the degradation of higher than 70% after 10 d incubation (Fig. 8). Among them, the degradation of C18 was the highest, which was 81.33%, and the degradation rate of C36 was the lowest, which was 7.65%. To date, several thermophilic hydrocarbon-degrading microorganisms were isolated from oil wells, as follows: a strain of *Acinetobacter* sp., which is capable of degrading long-chain n-alkanes (C13-C44)^43^; *Dietzia* sp. DQ12-45-1b, which can utilize hydrocarbons with chain-lengths from C6 to C40^23^; and a strain of *Geobacillus* sp. and *Rhodococcus* sp., which are able to degrade n-alkanes up to C36^22,44^. To our knowledge, our study is the first report on a *B. licheniformis* strain that can degrade long-chain alkanes (C36+) at high temperature.

### Viscosity reduction of crude oil

The high viscosity of crude oil, especially in heavy oil reservoirs, is one of the major reasons that lead to the low oil recovery efficiency^2^. After treatment with DM-1 strain for 15 days, the crude oil viscosity decreased by 40%, from 110 mPa·s to 66 mPa·s. This indicates that strain DM-1 has potential for enhanced oil recovery by reducing the oil viscosity.

### Oil recovery in the sandpack model

Sand pack flooding tests were carried out at simulated oil reservoir conditions to determine the *in-situ* MEOR efficiency of DM-1. The injected water and crude oil used in the tests were collected from the oil reservoir from which strain DM-1 was isolated. Moreover, a nutrient solution contained molasses (2%, w/v) was injected into the cores as the cosubstrate to improve EPS production and increase oil recovery. Results of the oil recovery were shown in Table 1. The oil recovery efficiency after the first water flooding was approximately 43%, which was attributed to volumetric sweep efficiency that occurred in the process of water injection^14^. After the first water flooding, bacterial culture and nutrient solution were injected to the cores, and the oil recovery efficiency increased by *in-situ* bioaugmentation with DM-1 was examined.The results revealed that for the nutrients flooding, 6.9% oil recovery efficiency was achieved, while for the *in-situ* test with strain DM-1 and nutrients (BH medium and 2% molasses), the recovery was 19.2% of OOIP. In contrast, it only increased by 3.8% with DM-1 injection only.

**Table 1:** The parameters and results of the core-flooding test.

Tested project	Porosity (%)	Permeability (μm^2^)	Oil recovery efficiency (%OOIP)		
			First water flooding	Subsequent water flooding	Recovery efficiency
Brine	23.1	2.98	42.6	42.8	0.2
DM-1+ Nutrients	23.1	2.96	42.6	61.8	19.2
DM-1	23.2	3.03	42.8	46.6	3.8
Nutrients	23.1	2.98	43.2	50.1	6.9

The oil recovery efficiency was within the range described in previous studies (Table 2).

**Table 2:** Comparison of *in-situ* MEOR of different strains.

Strain	Origin	Hydrocarbon utilization	Product	Test	Conditions	Oil recovery efficiency	Reference
*Bacillus licheniformis* DM-1	A deep subterranean oil reservoir	Crude oil (C12-C36)	EPS	Sand pack flooding	45 °C, 7 days	3.8%–19.2%	This study
An engineered strain constructed from an EPS-producing strain and a thermophilic strain	Oil field	ND	EPS	Sand pack flooding	40 °C, 7 days	11.3%	^16^
*Bacillus licheniformis* BNP29	Northern German oil reservoir	ND	EPS	Core flooding	47 °C, 20 days	9.3%–22.1%	^15^
Consortium of *Clostridium* spp.	Formation water from oil wells	ND	EPSs, acids, surfactants	Sand pack flooding, core flooding	40 °C, 14 days	26.7%, 10.1%	^46^
Consortium of *Brevibacillus* spp.	Brine samples from a high salinity petroleum reservoir	Crude oil	Lipopeptide	Sand pack flooding	45 °C, 7 days	7.03%–10.15%	^47^
Consortium of *Enterobacter cloacae* and *Pseudomonas* sp.	Crude oil-contaminated soil	Crude oil	Biosurfactant	Core flooding	40 °C, 7 days	19.1%–48.8%	^24^
*Geobacillus stearothermophilus* A-2	Produced water of Dagang petroleum reservoir	Crude oil (C12-C36)	Bioemulsifier	Core flooding	69 °C, 15 days	6.8%	^22,48^
Consortium of *Thermoanaerobacter* spp.	A Mexican oil reservoir	Heavy hydrocarbon fractions	Biosurfactant, acids, gases	Core flooding	70 °C, 20 days	19.48%	^49^
Consortium of *Thermoanaerobacter* spp.	Formation fluids from different oil wells	ND	Biosurfactant, volatile fatty acids	Core flooding	70 °C, 10 days	19.2%	^50^
Consortium of *Methanothermobacter* sp. and *Thermoanaerobacter* sp.	Formation fluids from different oil wells	Crude oil	Volatile fatty acid, gases	Core flooding	70 °C, 10 days	15.49%	^51^
*Bacillus subtilis* M15-10-1	Produced water of a water-flooded petroleum reservoir	—	Lipopeptide	Core flooding	40 °C, 7 days	16.71%	^52^

ND, not determined; (−), Negative.

Our results indicated that the *in-situ* bioaugmentation with DM-1 can efficiently recover the trapped oil under harsh conditions similar to oil reservoir.

The improvement of oil recovery efficiency may be associated with the following oil displacement mechanisms of strain DM-1. First, strain DM-1 can produce an EPS with high viscosity, which can lower water-to-oil mobility, thus improving the sweep volume of the injected water. Second, strain DM-1 could degrade heavy oil fractions and decrease the viscosity of the crude oil. Upon exhaustion of the injected carbon source (molasses), DM-1 can degrade and utilize crude oil, thereby increasing the quantity of light crude oil. This lighter oil has low viscosity and better fluidity and is therefore easy to be recovered from producing well^45^.

## Conclusion

An indigenous strain (*B. licheniformis* DM-1) showing superior properties required for *in-situ* MEOR was isolated from a mature oil reservoir in Dagang oilfield of China. *B. Licheniformis* DM-1 was able to utilize petroleum hydrocarbons and molasses for production of EPS under oil recovery conditions. The EPS from *B. Licheniformis* DM-1 was a glycoprotein with viscosifying and shear thinning properties. *B. licheniformis* DM-1 could degrade a wide range of n-alkanes with chain-lengths of C12–C36. Viscosity measurement shows that strain DM-1 reduced the viscosity of the crude oil by 40%. The results of sand pack flooding experiments showed that the *in-situ* bioaugmentation with strain DM-1 enhanced oil recovery by 19.2%, which highlighted its potential application in oil exploration. However, additional experimentations, such as further checking the EOR potential by using core plugs from a given oil field, monitoring the change in microbial communities during EOR via high-throughput sequencing, and evaluating problems caused by the growth of notorious sulfate-reducing bacteria, are necessary to support field trials.

## Supplementary information

Supplementary information.
