# Supplementary material for: A novel exopolysaccharide-producing and long-chain n-alkane degrading bacterium Bacillus licheniformis strain DM-1 with potential application for in-situ enhanced oil recovery
Source: Sci Rep. 2020 May 22;10:8519. doi: 10.1038/s41598-020-65432-z (PMC7244480; doi:10.1038/s41598-020-65432-z)
Supplement: Supplementary file 1 — Supplementary information. [file 41598_2020_65432_MOESM1_ESM.doc]

A novel exopolysaccharide-producing and long-chain n-alkane degrading bacterium *Bacillus licheniformis strain* DM-1 with potential application for *in-situ* enhanced oil recovery

Yanhui Fan^1,3^**·** Jun Wang^1,2,3^ **·** Chunming Gao^1,2^ **·**Yumiao Zhang^1,2^ **·** Wen Du^1,2^

* Jun Wang

[ivywangjun@163.com](mailto:ivywangjun@163.com)

^1^  College of Biological and Environmental Engineering, Binzhou University, Binzhou 256600, PR China

^2^  Shandong Engineering and Technology Research Center for Wild Plant Resources Development and Application of Yellow River Delta, Binzhou 256600, PR China

^3^ Shandong Provincial Key Laboratory of Eco-environmental Science for Yellow River Delta, Binzhou 256600, PR China

DM-1(DQ539620)

*Bacillus licheniformis* (LR134165)

*Bacillus paralicheniformis* (CP033389)

*Bacillus subtilis* (AB862124)

*Bacillus glycinifermentans* (CP023481)

*Bacillus sonorensis* (HM191249)

*Bacillus megaterium* (KY085976)

*Azotobacter chroococcum* (LN874286)

*Geobacillus stearothermophilus* (KR999931)

*Bacillus flexus* (KP419968)

*Bacillus paraflexus* (NR 135732)

100

48

91

100

54

60

82

0.005

**Fig. S1.** Phylogenetic tree based on 16S rRNA sequences demonstrating the relationship of *B. licheniformis* DM-1 to the closest related *Bacillus*


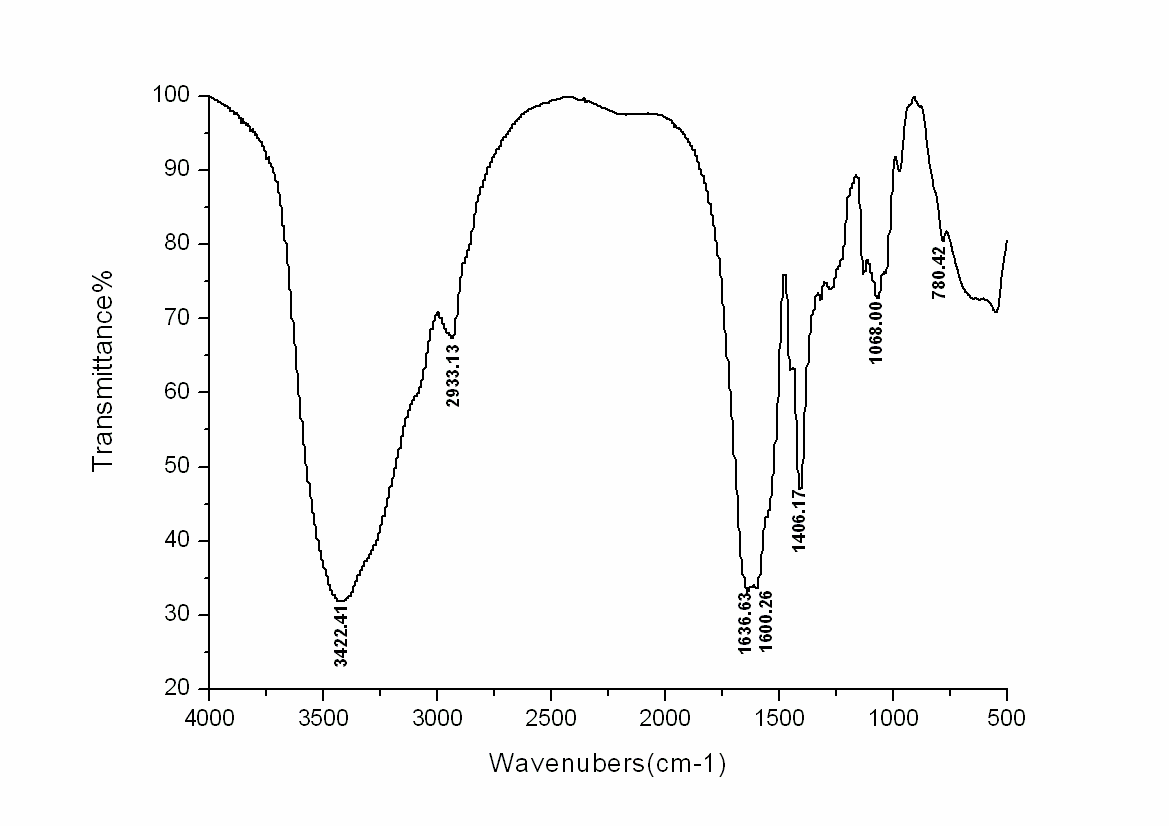


**Fig. S2.** Fourier transform-infrared spectrum of the purified DM-1 EPS


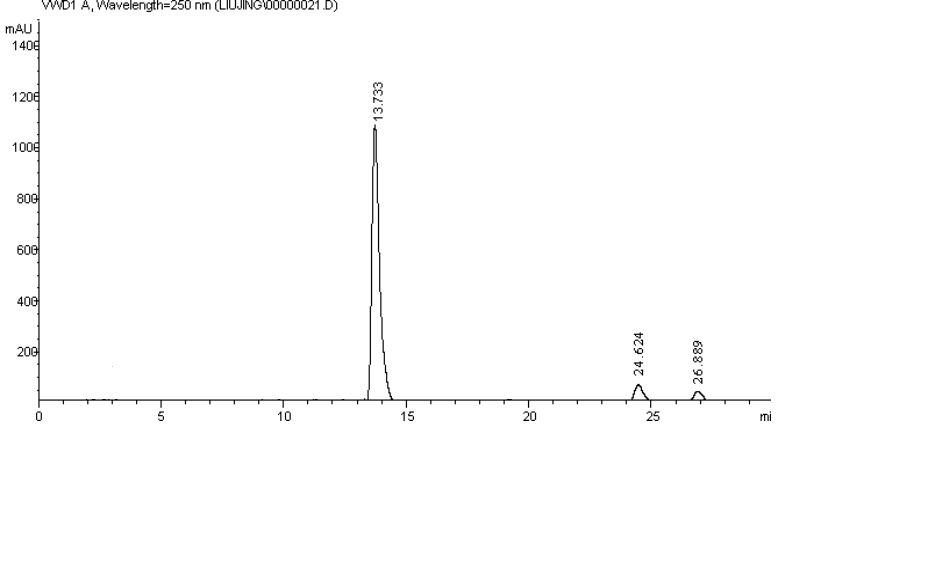


1

2

3

**Fig. S3.** Monosaccharide analysis of the DM-1 EPS samples by HPLC. The chromatographs of the EPS from show peaks for (1) mannose, (2) glucose, and (3) galactose.


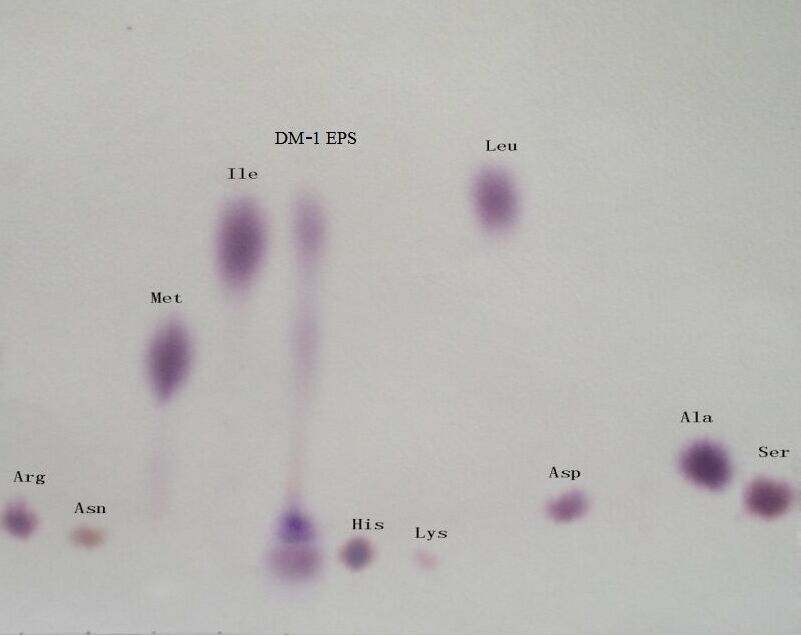


**Fig. S4.** Paper chromatography of amino acids from the DM-1 EPS

**Table S1 Characteristics of the formation brine**

| **Parameter** | **Concentration(mg/l)** |
| --- | --- |
| Na^+^ | 7615 |
| Ca^2+^ | 369 |
| Mg^2+^ | 107 |
| Cl^-^ | 12787 |
| NH_4_^+^ | 104 |
| P | < 0.04 |
| Fe | < 0.1 |
